# Neoliberalism and Radical Rights: On the Work and Theory of Law and Organising

**DOI:** 10.1007/s11196-022-09931-4

**Published:** 2022-09-05

**Authors:** William Fay

**Affiliations:** University of Adelaide, Adelaide, Australia

**Keywords:** Law, Legal rights, Neoliberalism, Critical legal studies (CLS), Rights discourse, Community organizing, Tenant unionism, Labour law

## Abstract

This article aims to contribute to scholarship regarding the critique of rights through the examination of the role of rights discourse in the furtherance of what is generally termed ‘community organising’—in particular, tenant organising—in the social and political context of neoliberalism. Conceptions of neoliberalism advanced in the work of David Harvey, Michel Foucault, Wendy Brown, and Bonnie Honig are synthesised to explicate the material and discursive role of law in the maintenance and furtherance of the neoliberal project. The article assesses the left-legal critique of rights presented primarily through the Critical Legal Studies Movement alongside the role of legal practice and legal discourse in countering neoliberalism. The article argues that rights claims concerning collective organisation, such as those commonly afforded to workers and trade unions, present a unique exception to the left-legal critique of rights in providing the means by which oppressive social relations may not only be remedies, but overcome. The works of Chantal Mouffe and of Roberto Unger are instructive in this regard and are placed in conversation with theories of community and labour organising. The article concludes by sketching the application of this conception of organising rights to the problem of housing and tenants’ rights under neoliberalism.

[I]n reality, the crisis we just experienced was waking from a dream, a confrontation with the actual reality of human life, which is that we are a collection of fragile beings taking care of one another, and that those who do the lion’s share of this care work that keeps us alive are overtaxed, underpaid, and daily humiliated, and that a very large proportion of the population don’t do anything at all but spin fantasies, extract rents, and generally get in the way of those who are making, fixing, moving, and transporting things, or tending to the needs of other living beings. It is imperative that we not slip back into a reality where all this makes some sort of inexplicable sense, the way senseless things so often do in dreams.

David Graeber, ‘After the Pandemic, We Can’t Go Back to Sleep’^1^

All the world that's owned by idle drones is ours and ours alone.

We have laid the wide foundations; built it skyward stone by stone.

It is ours, not to slave in, but to master and to own.

             Ralph Chaplin, ‘Solidarity Forever’

## Introduction

This article argues that the variety of inequalities exacerbated and broadcast by the COVID-19 crisis can be broadly characterised as features of neoliberal governance and political economy [59, p. 215]. The depth of these schisms is evident in the rapidly growing precarity of housing, inaccessibility of homeownership, and rising homelessness [51, pp. 15–8]. This has resulted in movements advocating for a cancellation of rents and evictions, for example those seen in Australia, the United Kingdom, and the United States [71, pp. 93–8; 86; 83]. The urgency of the COVID-19 crisis has resulted in temporary freezes and reductions in rent,^2^ stays on evictions,^3^ and provision of emergency accommodation for people experiencing homelessness [90, 91]. These actions have shown the ways in which legislative and governmental action can swiftly remedy the immediate concerns of renters, low-income workers, and those experiencing homelessness. As these measures have begun to be rolled back, and the COVID-19 pandemic appears to have exacerbated, not upturned, this neoliberal status quo [59, pp. 219–23], tenants, workers, and others who have been the victims of neoliberalism’s ‘class war from above’^4^ find themselves in an entrenched state of uncertainty.

The neoliberal project, for the purposes of this article, will be defined primarily by a certain hegemonic conception of political and legal community that elevates the entrepreneurial individual and capitalist market relations, as well as a concomitant conception of the role of the state. The emergence of the global neoliberal order can be seen as facilitated and promoted by the law,^5^ with governmental regimes utilising various legal mechanisms and, of greatest interest for the purposes of this research, forms of legal rhetoric—particularly with regard to rights—to naturalise certain political, economic, and social relationships, and delegitimise others. Neoliberalism is often conceptualised as an overriding ‘political logic’.^6^ As Gerwal and Purdy describe, neoliberalism can be understood as an interconnected array of propositions, particularly in legal argument and scholarship, that ‘defin[e] and regulat[e] market relations in ways that insulate them from democratic politics’ [55, p. 14]. In this way, neoliberalism is not merely the use of legal mechanisms to implement pro-market economic policy, but is best understood as an ideological position (ideological in that it represents a totalising worldview with a certain conception of political ‘community’) in the struggle between ‘democratic imperatives’ on one hand, and ‘capitalist imperatives’ on the other [55, p. 23]. In this regard, neoliberalism does not simply allow market arrangements to emerge and flourish on their own terms, but utilises the power of the state—and, therefore, the law—to create market relationships where they otherwise might not exist (through, for example, privatisation).

In the housing context, the market-forward rationale of neoliberalism can be seen in the rejection of the ‘social project’ of homeownership and the role of the state in the direct, universal provision of housing [87; See also 36]. Instead, housing has been defined by the ‘relentless logic of commodification’ that is a hallmark of the neoliberal project [18, pp. 233–44]. Any endeavour that that aims to counter the hegemonic power of neoliberalism must primarily be concerned with broadening the range of political and institutional arrangements deemed possible within public discourse. It is in this way that I will argue legal scholarship and rhetoric, as well as legal mechanisms, that promotes *collective*, *participatory* organising may be usefully deployed.

The relationship between legal mechanisms and rhetoric, and the process of social change has been a vexed issue for commentators and practitioners interested in the pursuit of a ‘left’ legal project. It is in this context that the relationship between law, and social movements and methods of community organising is to be explored. This research aims to explore and advocate for a regime of legal protections for the collective organisation of private and public housing tenants, particularly in the Australian jurisdictional context, and liberal democracies of the Global North more generally. First, this project will find its theoretical bases in the Critical Legal Studies (‘CLS’) critique of rights,^7^ drawing also upon the origins of this critique in Marxist legal analysis. In so doing, ‘organising rights’—such as the protections provided to workers wishing to collectively organise—will be distinguished from traditional political and civil liberties. Second, the necessary components of a counter-hegemonic politico-legal project that aims to expand democratic possibility in the face of neoliberalism will be discussed, drawing particularly from the work of legal theorist Roberto Unger and political theorist Chantal Mouffe. The article will then conclude by considering, in the context of this politico-legal counter project, reforms aimed at promoting organising through the securing of organising rights, finding analogy in existing legal protections for the organisation of workers and historical instances of collective organising.

The foundational position of this article is that, unlike other rights forms, organisational rights serve two unique purposes, and these purposes provide a kind of harmonisation of rights mechanisms with the critical analyses forwarded by CLS scholars. This harmonisation presents organisational rights as a means by which traditional liberal ‘rights discourse’ may be transcended. First, organisational rights allow for not only the creation and enunciation of alternative visions of the formation of society and its institutions, but also for the development of countervailing social, political, and economic power structures that cement these visions and provide a means for their material establishment. Such rights act to revivify *agonism* in democracy and reaffirm the importance of struggle in democratic participation. The legal scholarship of Roberto Unger regarding the necessity of such alternative visions will be instructive in this regard [1, pp. 405–7; 69, p. 7]. Second, organisational rights serve a variety of practical and mechanical legal purposes in fostering and protecting collective organisation. This gives organising rights a kind of self-reinforcing political and legal function in that the provision of such rights can fuel the organic organisation of oppressed groups in ways that other rights forms cannot, and thereby help to create the social and political conditions for their full enjoyment.^8^ The scholarship drawn upon throughout the following analysis varies in its territorial origin—although being heavily influenced by the US CLS movement and the European Marxist tradition—however, to the extent that this article presents avenues for legislative reform in Part V, it is bound to the Australia jurisdiction. Despite this territorial limitation, the primary observations regarding the role of rights mechanisms and rhetoric in a left-legal project are of general importance and applicability to capitalist liberal democracies of the global north emerging from, or battling with, the impact of neoliberal governance—although these terms are themselves subject to significant political and scholastic contestation.

In sketching a framework for the securing of organising rights for tenants, cues will be taken from existing protections in Australian labour law, as well as the emergent field of ‘movement lawyering’ to consider the ways in which the methods advocated by academics and practitioners in the ‘movement law’ tradition create analogy with existing law concerning labour organising and provides indicators of appropriate legislative reform.^9^ The article will then conclude with a consideration of these reforms within the context of the theoretical and material goals of a broader left-legal project.

## Neoliberalism and The Limitation of Democratic Possibility

Neoliberalism, as a concept utilised across various fields of social theory, is notable not only for its ability to explain and encompass the variety of ills that afflict the current economic and socio-political moment, but also for its contested and varied definitions throughout the literature. Despite the lack of detailed consensus regarding what exactly neoliberalism is and how it manifests, there is a somewhat loosely defined policy program to which neoliberal governments and actors are generally agreed to adhere [11, pp. 17–8]. This program includes the privatisation of previously publicly owned resources and services, deregulation of industry, taxation policy aimed to entice foreign investment capital, regimes of free trade, increased economic financialization,^10^ and limitations upon labour unions and the rights of workers to act collectively [10, pp. 17–8]. Moreover, the pursuit of market freedom requires the state to take an active role in the creation of market relationships where they otherwise would not exist [28, p. 42; See also 26]. Behind this suite of economic imperatives sits a certain ideological foundation by which ‘human well-being can best be advanced by liberating individual entrepreneurial freedoms’ and the role of the state in the regulation of economic affairs and the advancement of any conception of the ‘common good’ is accordingly limited [26, p. 2]. This abandonment of non-individualised, normative judgement is well articulated by Paul Babie and Michael Trainor, writing that neoliberalism ‘is good at [economic] choice, but not good at all at telling us what is a good or a right or just choice.’ [7, p. 134].

Two primary conceptions of neoliberalism will be considered in turn. First, the Marxian class power analysis, exemplified in the work of David Harvey, and second, the Foucauldian rationality analysis, synthesised with the Marxian approach by Wendy Brown. Although offering vastly different foci in their analysis of neoliberalism, these conceptions, taken together, provide a fulsome account of both the discursive and material constitution of neoliberalism. Regard will be given to the work of Bonnie Honig in utilising object-relations analysis to describe the development of citizenship and political possibility, and the threat posed by neoliberalism to these phenomena. A view of neoliberalism will be advanced that acknowledges the vital class dimension of both neoliberalism and any viable counter-neoliberal project, whilst also acknowledging the crucial and novel ways in which hegemonic power is maintained and advanced under neoliberalism through the remaking of subjectivity and the limitation of democratic possibility. Parts III and IV will then expand upon the description of this counter-neoliberal project, emphasising the importance of law and legal discourse in its development. Part V will begin to apply this framework in providing avenues for reform.^11^ Part VI will conclude by summarising the instrumental advantages of organising rights in counter-neoliberal project and the rhetorical advantage of such rights, using the unifying lens of ‘semiotic indeterminacy’.

### Neoliberalism as Class Politics

Whilst not explicitly taking up the Foucauldian emphasis on the impacts of neoliberalism on subjectivity, Harvey views neoliberalism as being interpreted ‘either as a utopian project to realize a theoretical design for the reorgani[s]ation of international capitalism or as a political project to re-establish the conditions for capital accumulation and to restore the power of economic elites.’ [26, p. 19] Harvey is firmly of the view that neoliberalism is best conceptualised as a political project. To Harvey, the post-war period in which the interventionist welfare state expanded, and Keynesian monetary policy was deployed to regulate the boom-bust cycle of free-market capitalism—referred to as ‘embedded liberalism’ [26, p. 11]—represented a kind of ‘class compromise’ between the interests of organised labour and capital [26, p. 10]. This compromise saw the owner class receive a ‘stable share of an increasing pie’ in exchange for robust social welfare provisions and state leadership in matters of economic growth, overriding the traditional battle between the interests of labour and capital. The ‘stagflation’ crisis of the 1970s, characterised by high levels of inflation, coupled with high unemployment and low economic growth, put the position of capital under this arrangement in a state of precarity. Harvey writes that ‘when real interest rates went negative and paltry dividends and profits were the norm, then upper classes everywhere felt threatened.’ [26, p. 15].

The Marxian view thus explains the emergence of neoliberal governance as a function of the waning power of capital and the crisis of profitability presented by ‘stagflation’ that sought to ‘disembed capital from [the] constraints’ of the post-war compromise [26, p. 11]. On this view, neoliberalism was ‘from the very beginning a project to achieve the restoration of class power.’ [26, p. 16] Contrary to the view of neoliberalism’s champions, the class politics interpretation argues, therefore, that neoliberalism advances no genuine, positive vision, but it exists primarily ‘as [an ideological] mask for practices that are about the maintenance, reconstitution, and restoration’ of this class power [26, p. 188]. This disingenuity and ideological incoherence, for Harvey, will be the process by which neoliberalism facilitates its own downfall, as the economic inequalities that are hallmarks of neoliberal political economy deepen, and the façade of ‘freedom’ used to validate the neoliberal order become increasingly tenuous [26, pp. 188–9]. Harvey therefore reasons that the ‘profoundly anti-democratic nature of neoliberalism… should surely be the main focus of political struggle’ against neoliberalism [26, p. 105]. Reinforcing the Marxian disposition of Harvey’s approach, the nature of neoliberalism as a class-based political project requires the engagement of an anti-neoliberal project in class struggle.

Harvey’s analysis concludes that what is necessary to overcome neoliberalism is not only a revivification of class politics, but a revivification of democracy that seeks ‘to bring back the demands for democratic governance and for economic, political, and cultural equality and justice’ [26, p. 206]. However, to do so is not to romanticise embedded liberalism, but to acknowledge both its successes and failures such that notions of equality and justice are ‘reinvented to deal with contemporary conditions and potentialities’ [26, p. 206]. It is worth noting that positioning the content of claims for reinvigorated democracy and deepened equality as contingent upon ‘contemporary conditions and potentialities’ may be seen to highlight the Marxian—or, more appropriately, materialist—nature of Harvey’s analysis, in that the political content of claims for equality are defined by the material conditions and relationships in which they arise. The role of law, on the Marxian view, is largely peripheral to class struggle and, as will be further discussed, evidences the strict focus of the Marxian approach on objective material conditions. This strict view fails to adequately address the discursive and subjective dimensions of neoliberalism and the role of such dimensions in countering neoliberal hegemony. Despite the deficits of a strictly materialist, Marxian analysis, the role of rights discourse in engaging this struggle is of significant importance to Harvey. This analysis of struggle against neoliberalism will be considered, in concert with the Foucauldian conception, in the mapping of a politico-legal counter project in Parts IV and V.

### Neoliberalism as Rationality and Governmentality

The work of legal scholar Wendy Brown, drawing on Michel Foucault, conceptualises neoliberalism beyond simply a new development in class politics as a remaking of subjectivity by which market logic exists as a hegemonic and totalising force of reason internal and external to democratic subjects. As will be discussed, Foucault describes the neoliberal vision of the subject as *homo oeconomicus—*economic man. Foucault provides a genealogy of the problematic of *homo oeconomicus* as beginning with the emergence of enlightenment liberalism and the introduction into western thought of the vision of an atomistic subject ‘who appears in the form of a subject of individual choices which are both irreducible and non-transferable’, with these choices forming the source of the subject’s ‘interest’ [19, p. 272]. Foucault places this ‘subject of interest’ in tension with the ‘juridical will’ in that this view of the subject is incompatible with the foundational ‘social contract’ by which the legal subject is traditionally viewed to be created [19, p. 273]. Although it may initially appear that the ‘juridical will’ is created by the calculation of such interests in the formation of the basic ‘social contract’, in actuality ‘interest constitutes something irreducible in relation to the juridical will.’ [19, p. 274] To Foucault, the juridical subject of right necessarily requires the splitting and subordination of certain aspects of itself in relinquishing certain natural rights to a sovereign. This view of the juridical subject—*homo juridicus—*is therefore contrasted with *homo oeconomicus* in that *homo oeconomicus* is ‘never called upon it relinquish [its] interest’ and thereby exists as a ‘heterogenous structure’ to *homo juridicus* [19, pp. 275–6].

Foucault places the problematic of *homo oeconomicus* in direct contradiction with what Harvey described as neoliberalism’s ‘utopian project… for the reorgani[s]ation of international capitalism’ [26, p. 19]. This utopian view, contrary to Harvey, takes the rhetorical and theoretical claims of proponents of neoliberalism as genuine. Proponents position the neoliberal subject as a liberated individual, released from the coercion and restrain of governance and allowed to freely pursue its interest. Foucault argues, however, that economic rationality requires that *homo oeconomicus* ‘responds systematically to systematic modifications artificially introduced into the environment.’ [19, p. 270] This uniform method of engagement with the world leads *homo oeconomicus* not to freedom and emancipation, but toward a state of ‘emine[nt] governab[ility]’ [19, p. 270]. This does not replace juridical governance with freedom, but with an economic governance of the market. On this account, the neoliberal subject is not ‘an atom of freedom in the face of all the conditions… of a possible government… [but] a certain type of subject who precisely enables an art of government to be determined according to the principle of economy’ [19, p. 271]. This process by which the governing rationality of neoliberalism is internalised, and thereby reshapes us as subjects, is integral to the Foucauldian conception of neoliberalism and, moreover, to Brown’s extension of the idea of *homo oeconomicus*.

Brown expands Foucault’s conception of neoliberalism by detailing the way in which the emergence of *homo oeconomicus* casts citizens as entrepreneurial monads—units of ‘human capital’—whose sole purpose is found in the enrichment of said capital in accordance with its placement in the ‘marketplace’ [10, pp. 36–41]. Moreover, Brown draws upon the neo-Marxian view previously discussed to understand the nature and development of ‘actually existing neoliberalism’ and the political movements that propel it [10, p. 21]. Brown synthesises the Foucauldian approach—characterised as ‘revealing the extent to which capitalism is not singular and does not run on its own logics, but is always organi[s]ed by forms of political rationality’—and the neo-Marxist focus on economic policy, institutions, and class dynamics not as diametric opposites as described by Harvey, but as salient descriptions of various facets of the neoliberal project [10, pp. 20–1]. Brown’s analysis also draws upon the political movements that have propelled neoliberalism and their devolution towards authoritarian, xenophobic, and intensely moralistic forms of right-wing politics.^12^ Of primary interest to this analysis is Brown’s development of neoliberalism as transforming the democratic subject and the particular role of law and legal discourse in this process.

The neoliberal subject, conceptualised as human capital is ‘at once in charge of itself, responsible for itself, yet an instrumentalizable and potentially dispensable element of the whole.’ [10, p. 38] Brown here draws on Foucault’s contrast between *homo juridicus* and *homo *oeconomicus in emphasising the ways in which the neoliberal conception of the subject undermines the foundations of the social contract and dispenses with the a priori assumption of human equality necessary for the functioning of democracies. Moreover, what is unique in neoliberalism, to Brown, is the ability of market rationality to extend far beyond the sphere of commerce into all other aspects of human experience. Where liberalism commanded that the fullness of the human experience required ‘pursuing our own good in our own way, so long as we do not attempt to deprive others of theirs, or impede their efforts to obtain it’ [44, p. 19], under neoliberalism we are ‘everywhere *homo oeconomicus* an only *homo oeconomicus*.’ [10, p. 33] Brown describes this as ‘economization’, the process by which economic rationality extends both informally, in the minds of subjects, and formally, through the actions of the state (for example, privatisation), to aspects of life to which it was previously foreign. It is in this way that neoliberal rationality secures its totalising and *hegemonic* power, disseminated throughout the citizenry to such a degree that it delineates the confines of personal and political possibility that is then internalised and comes to limit not only the policy prescriptions to which we subscribe, but the very nature of our subjectivity. In this sense, Brown’s conception is largely inescapable, coming to define the very way in which we might approach neoliberalism as a social and political problem and thereby limiting our ability to imagine—let alone realise—a world beyond it.

Contrary to Brown’s view of neoliberalism as a near-inescapable hegemonic force, Bonnie Honig offers a conceptualisation of democratic citizenship that, whilst not incompatible with the analyses heretofore discussed, provides the immediate possibility of developing citizenship beyond and in opposition to neoliberal rationality. Honig’s analysis is grounded in object relations psychoanalysis, arguing that in much the same way that objects are viewed as central to infants developing an ability to engage with the world as an external reality, so too are ‘public things’ (for example common spaces, infrastructure, services) necessary for the development of a collective sense of citizenship [29, p. 15]. It is in this way that, to borrow the Foucauldian term, *homo juridicus* emerges through a form of social contract that ‘depend[s] partly upon objects to help collect diverse citizens into self-governing publics… invested with a sense of integrated subjectivity, responsibility, agency and concern.’ [29, p. 17] Honig notes the considerable degree to which neoliberal practice undermines this phenomenon by privatising and commodifying public things and thereby removing them of their power in the collective imaginary. However, in applying this approach to citizenship formation in critique of Brown, Honig sees the ‘constitutive necessity’ [29, p. 15] that public things hold to democratic life as indicative of their ability to provide avenues to resist and transcend neoliberal rationality [29, pp. 20–2]. Where Brown presents neoliberalism as totalising and hegemonic, Honig provides a useful avenue for consideration regarding how to preserve—and even expand—the vestiges of an open, democratic, and egalitarian vision that exist in our neoliberalising society, as will be discussed further in Part IV. Taking together these understandings of neoliberalism and the integral and constitutive role of law and legal discourse in the neoliberal project, it is useful to now consider the ways in which the law has previously been conceptualised for the purpose of social change.

## What has Been Done: Left-Lawyering and the Critique of Rights

The relationship between legal mechanisms and rhetoric, and the process of social change has been a vexed issue for commentators and practitioners interested in the pursuit of a ‘left’ legal project. As the previous discussion of the role of the law and legal discourse in legitimating and furthering neoliberalism illustrates, the legal institutions that define and constrain our political communities are not fertile ground for the advancement of radical politics. Moreover, the law and the legal profession as they presently exist are often complicit in the kind of hierarchy and institutions radical theorists and practitioners seek to challenge. To properly articulate the ways in which tenant unionism may counter neoliberal discourses and practices surrounding the provision of housing, it is important to understand the ways in which this tension between law and social change has been understood previously. This tension, and the critique it inspires, forms the foundation of the left-legal critique of the prevailing instrument of progressive reformers—rights. The critique of rights is most cogently enunciated by scholars in the CLS tradition. Therefore, in seeking to understand the ways in which radical practitioners and reformers should approach rights—especially, for the purposes of this research, organising rights—regard must be given to the central themes present in what can broadly be described as the left-legal critique of rights. The intellectual and political heritage of the critique of rights is found first in the seminal work of Karl Marx and the articulation of a materialist analysis of law, informed by a more general critique of liberalism. Further, the traditional materialist critique is complicated by the intervention of CLS scholars in seeking to understand the role of law as both the product of social relations and an arena within the ‘social totality’ in which battles for social justice may be fought.

### Marxist Origins

The left-legal critique of rights finds its origins in the work of Karl Marx, principally in his early essay ‘On the Jewish Question’ (‘OJQ’). Although more detailed discussion is beyond the scope of this research, it should be noted from the outset that significant portions of this essay are widely regarded as anti-Semitic and contain anti-Semitic characterisations of Jews and Judaism. Moreover, Marx’s personal relationship with Judaism and Jewish emancipation is of considerable scholastic debate.^13^ For the purposes of this analysis, OJQ will be discussed for its application to the critique of liberal rights and, resultantly, the state within Marx’s work. Israeli political scientist Yoav Peled describes OJQ as operating within the contemporaneous debate regarding the status of Jewish citizens and seeking to ‘shift… the debate over Jewish emancipation from the plane of theology… to the plane of sociology… [and] present a powerful case for emancipation while, at the same time, launching [Marx’s] critique of economic alienation’ [52, p. 463]. Peled’s distinction between theological and sociological analysis clearly indicates the distinctive materialist quality of Marx’s analysis—in stark contrast to idealist interpretations of historical development—that informs Marx’s critique of 19^th^ Century liberalism.

Both idealism and materialism hold that history develops *dialectically*, that is through the conflict and resolution of contradictory positions, however idealists view this development as occurring between forms of being and consciousness, or ideas, whereas materialists view this development as occurring through changes in material conditions—that is, economic and productive realities. In this way, Marx’s focus upon the development of objective material conditions and productive forces informs the view taken in OJQ that the provision of traditional liberal rights, or *political emancipation*, is insufficient for the true emancipation of both the individual and the collective. Marx’s critique of liberal rights centres around two primary contentions—first, that liberal rights do not in and of themselves constitute proper or full freedom for the oppressed and marginalised, and, second, that rights themselves are individualising and, to this extent, inadequate means for achieving material equality. These contentions will now be outlined in turn.

Marx outlines the limitations of liberal rights first by differentiating between *political emancipation*, that is the provision of formal equality between citizens in liberal democracies, and true human emancipation, realised through material equality. This distinction is drawn sharply to criticise the ideals of the French and American revolutions, enunciated in the Declaration of the Rights of Man and of the Citizen [39, pp. 41–2]. In the context of religious liberties, political emancipation, although noted to be a ‘great progress’ [39, p. 35], does not extinguish the role of religion in society. Marx observes that political emancipation on grounds of religion instead relegates the influence of religion to the private sphere, where, although the state may regard itself as unentangled from matters of religion, ‘the *immense majority* of people continue to be religious [and they] do not cease to be religious by virtue of being religious in *private*.’ [39, p. 32] In this way, liberal rights in actuality represent a freedom of the state to ‘liberate itself from a constraint without man himself being *really liberated*’ [39, p. 32]. Political emancipation, therefore, does not seek to substantively challenge the forces of societal oppression, but merely partition this oppression away from public life [39, p. 35]. True human emancipation, therefore, is only achieved through the development of material relationships. Marx writes that political emancipation ‘is [merely] the final form of human emancipation *within* the framework of the prevailing social order.’ [39, p. 34] It is in this way that Marx’s analysis shifts from the ‘theological’ to the ‘sociological’ in analysing the material structures that give rise to social relations as opposed to analysing discrete individualised matters within a framework that presupposes these structures. The provision of liberal rights, to Marx, presupposes and naturalises certain social relations, in particular relations resultant from the institution of private property. Marx writes that, with the provision of liberal rights, the state ‘allows [these social relations] to act after their own fashion… Far from abolishing these effective differences, it only exists so far as they are presupposed…’ [39, p. 33].

Where the idealist approach sees the emergence of liberal rights as natural, intellectual phenomena, Marx sees such rights as the result of concrete historical processes, namely the transition between various modes of production and state organisation [38, p. 40]. The presupposition and naturalisation of liberalism Marx describes prefigures the function of neoliberal discourses of rights and individual freedom to justify and internalise certain power relations within citizens and limit democratic possibility as discussed in Part II. Moreover, Marx saw liberal rights as not only failing to advance true emancipation, but as advancing an inhumane, atomistic view of human nature and community.

Again engaging the Declaration of the Rights of Man and of the Citizen, Marx positions rights as necessarily competitive phenomena founded upon ‘the separation of man from man’, with liberty conceived only as ‘the right of such separation. The right of the *circumscribed* individual, withdrawn into himself.’ [39, p. 42] One’s possession of a right confers upon them an ability to exercise this right against all others. On this view, rights may only be exercised by individuals and therefore limits our conception of political and public life to the engagement of atomised individuals, without regard for our collective circumstance. This view fundamentally shapes both our experience and conception of social relations such that it ‘leads every man to see in other men, not the *realization* but rather the *limitation* of his own liberty’ [39, p. 42].

The compulsion of Marxian analysis to disengage with law—actual or inferred—is a significant site of development in subsequent left-legal scholarship. Although CLS scholarship, as will be further discussed, is informed significantly by materialist and neo-Marxian analysis, the CLS critique of rights maintains a full-throated critique of liberalism and traditional legal reformist dogma, whilst engaging the possibility of the advancement of a broader radical project through the law.

### Critical Legal Studies

The Critical Legal Studies (‘CLS’) movement of the 1970s and 80 s is characterised first and foremost by its varied and seemingly disparate iterations [58, pp. 121–2]. The CLS movement will be considered for these purposes a somewhat loose association of primarily US legal academics that sought to import the developments and ideals of the New Left era into the legal academy and legal practice [81].^14^ The overriding themes of the CLS movement which form the focus of this examination concern the critique of formalism and objectivism in legal reasoning [67, pp. 1–2] —that is the dissolution of the law/politics distinction found in liberal legalism—and the implications of this view for the critiques of rights and of liberalism. These themes will be explored to develop an approach to the problematic of legal rights and organisation for the reimagining of society, in particular a reimagining of the landlord/tenant relationship and the commodity status of housing, suitable to counter the hegemonic power of neoliberalism and its concomitant/constituent legal discourse.

#### CLS and Law

The CLS approach to legal analysis can be understood as founded upon the rejection of two key principles CLS scholars view as integral to traditional jurisprudential approaches. These are, first, the rejection of formalism, and, second, the rejection of objectivism. Formalism is taken broadly within CLS scholarship to refer to a ‘commitment to, and… belief in the possibility of, a method of legal justification that contrasts with open-ended disputes about the basic terms of social life, disputes that people call ideological, philosophical, or visionary.’ [67, p. 1] On the formalist view, legal doctrine is analysed, or discovered, through the application and development of ‘impersonal purposes, policies, and principles’ and is fundamentally non-political [67, p. 1]. CLS scholars instead embrace a *realist* approach that affirms the political and contested nature of legal reasoning. Objectivism, mirroring Marx’s description of the falsity of liberal legalism, is ‘the belief that the authoritative legal materials… embody and sustain a defensible scheme of human association [and] display… an intelligible moral order… [that is] not merely the outcome of contingent power struggles’ [67, p. 2]. Taking up, to varying degrees, the Marxist critique, CLS scholars invite us to view the law as fundamentally *indeterminate* and therefore the result of live political contest. The CLS movement, as will be further discussed, emerged from, and was integrally linked to, the social movements of the ‘New Left’ period and has always been deeply concerned with the link between legal theory and politico-legal praxis, utilising these jurisprudential principles to inform a progressive approach to lawyering in the pursuit of social justice [4, 67].

The way in which the CLS approach abandons the presupposition of an inherent logic of the law (a view held by CLS scholars that Duncan Kennedy places in direct opposition to Marx’s view of liberalism [32, p. 216]) allows for law to be seen beyond the simple base/superstructure dichotomy of traditional materialist interpretations of law, whilst acknowledging the inherently social and political nature of law. This approach emphasises the elements of Marx’s writing in OJQ that describe the mystifying and naturalising nature of law (that is, the process of *reification*^15^), whilst parting from the strictures of materialist analysis [23, p. 369]. The CLS approach allows the law to be understood, as Kennedy described, as ‘not simply “superstructural”’ but as ‘an aspect of the social totality’ [33, p. 10]. On this view, the law becomes one of many possible grounds for political contest. Kennedy writes that ‘[law] is part of the equation of power rather than simply a function of it, people struggle for power through the law… The outcomes of struggle are not preordained by any aspect of the social totality’ [33, p. 10].

Moreover, the legal system presents a means by which the prevailing social order, through the state, may maneuverer to legitimise itself against the majority population’s lived experience of alienation [23, p. 370]. Peter Gabel and Paul Harris outline the basic claim of the CLS approach to law as ‘that the very public and political character of the legal arena gives lawyers, acting together with clients and fellow legal workers, an important opportunity to shape the way that people understand the existing social order and their place within it.’ [23, p. 370] Just as the law may express the dynamics and hierarchies of our society and institutions, and operate as a hegemonic instrument, the fundamental indeterminacy of the law allows for it to be utilised for activist purposes, both as a means for securing material victory and as a kind of staging ground for public contest. The law, therefore, functions in large part to reify liberal capitalist relations, however it is the skills of a radical lawyer, on the CLS view, to determine when it is appropriate to engage with the legal system and in what way to formulate legal arguments for activist ends. This is referred to by Kennedy as the process of ‘legal work’ and forms, therefore, an important element of both theory and praxis in the pursuit of societal transformation [31, p. 785; 15 p. 7].

#### CLS and Rights

In applying the CLS approach to the critique of rights, Mark Tushnet outlines rights discourse as allowing one of four possible outcomes for legal and political contest:

1. A win in the courtroom that also represents a political win;
2. A win in the courtroom that represents a political loss;
3. A loss in the courtroom that represents a political win; and
4. A loss in the courtroom that represents a political loss [66, p. 25].

Tushnet’s articulation of the fundamental principle of the critique of right in CLS illustrates the way in which progressive legal projects must be sure not to mistake legal victories—of the kind akin to idealist legal developments outlined by Marx—for political or material victories. Unger describes this fundamental concern with the practical, material, and political impacts of legal development as a result of the CLS departure with objectivism previously outlined. Once objectivism is dispensed with ‘[l]egal reasoning… turn[s] into a mere extension of the strategic element in the discourse of the legislative jostling. The security of rights … fall[s] hostage to context-specific calculations of effect.’ [67, p. 3].

The result of this practical political calculus concerning rights claims has been the subject of much contention in subsequent critical legal theory. This concerns, ultimately, the individualising nature of rights discourse as outlined by Marx in OJQ. Wendy Brown argues that rights discourse ‘is a politics and it organi[s]es political space, often with the aim of monopoli[s]ing it’ [9, p. 461]. This outlines the *structural determinacy* of rights discourse—that rights discourse is by its very structure individualising and tied to the political project of liberal capitalism. On this view, rights discourse serves to displace any political project that may seek to use the discourse of rights to its own ends.^16^ Although rights may be *normatively indeterminate* in that they do not provide strict ‘right answers’, there is a ‘deeper determinacy at play’ by which critics argue rights can reduce the radical aims of otherwise effective political movements [93]. The utility of rights discourse is then determined by the extent to which it may be reworked to counter this structural determinacy and prove effective for a more radical project. As the forthcoming sections will argue, organising rights—as distinct from traditional, liberal civil and political rights—present a series of useful possibilities for overcoming this structural determinacy and advancing an expanded politics by providing material structures by which society may be transformed.

Kennedy further describes the legitimating role of rights claims in political discourse as ‘mediat[ing] between factual and value judgments’ in that a rights claim discursively allows one’s political preference to be asserted in objective, ‘legally correct’ terms [32, p. 185]. This appeals to the liberal notion of rights having somewhat of a natural quality, independent of political assertion, and echoes Marx’s observations in OJQ previously discussed [39, p. 33].^17^ Moreover, to Kennedy, liberal rights have a heavily counter-majoritarian role in the process by which intelligentsia seek to universalise a rights claim for which they ‘no longer believe (or never believed) that they represent the “will of the people”.’ [32, p. 190] Kennedy notes that this involves, from a left-legal perspective, the abandonment of majoritarian class analysis in which working class interests where championed over the interests of a minority owning class. The CLS attempt to throw off the atomising nature of rights discourse under liberalism, however, is complicated by the interjection of critical race theorists in developing an understanding of the protective function of rights for groups with less structural power when engaging with an oppressive or hostile social structure.

The work of critical race legal theorist Patricia J Williams regarding the critique of rights provides such a complication.^18^ Williams, acknowledging the central indeterminacy of rights and, recalling Marx’s description of the ‘egoistic man’ created by rights discourse, observes that, as a woman of colour—in particular, as an African-American woman—relationships formed by rights provide a ‘formal relation to the other’ without which she would be ‘[left] estranged’ [72, p. 148]. Grounded in her experience in the history of slavery and structural racism in the United States, in which individuals were not only conceived of in terms of their commercial output, but were themselves literally reduced to chattel, Williams emphasises the way in which the allegedly atomising and alienating nature of rights discourse provides to minority communities ‘freedom through the establishment of identity [and] the formulation of an autonomous social self’. Williams argues that the status of rights as ‘ends in themselves’ is largely irrelevant and that rights rhetoric is an effective method by which ‘change for the better must come (whether it is given, taken, or smuggled).’ [72, p. 149] Although change might be argued for in the ‘sheep’s clothing’ of stability through the deployment of rights rhetoric, the ends advanced by this rhetoric may themselves critique and even destabilise existing institutional arrangements [72, p. 149].

The addition of Williams’ commentary to the CLS critique of rights is useful in understanding the role of rights as both a ‘shield’ and imperfect ‘sword’ for minority groups. However, it must also be noted that this function as a ‘shield’, although ineffective and minimal as it may be, applies in less counter-majoritarian fashions also. The protective nature of rights against reactionary and oppressive forces can be seen in the protection of other, non-minority groups against groups that are more structurally powerful, although lesser with respect to sheer number. For example, the protection of organised workers against the unbalanced political and economic power of businessowners, imperfect though it may be, allows the for the persistence of trade unionism.^19^ These critical perspectives on rights for both political advancement and protection in the context of a radical and democratic counter-neoliberal project will now be considered.

## Reform (F)or Revolution: Organising Rights Distinguished

The integral link between the law—both as a mechanism for the structure and governance of our institutions, and as a mode of social, political, and economic discourse—and the validation and furtherance of the neoliberal project as discussed in Part II makes clear the necessity of the law in any attempt to challenge neoliberalism’s hegemony. The traditional critique of rights advanced by theorists of the left discussed in Part III illustrates the role of the law in the reification of the hierarchies and power relations our society produces. However, the contribution of CLS scholarship makes clear that an appropriate response to the oppressive utilisation of law is not to denounce law entirely as unworthy of political consideration, but to acknowledge law’s validity as an arena for social and political contest, and engage instrumentally with legal methods and discourses for activist purposes. In this part, the role of organising rights will be considered, in contradistinction to traditional liberal civil and political rights, as an appropriate basis on which a counter-neoliberal legal project should be advanced, and as a means by which the critique of rights previously discussed and the desire for material institutional transformation can be reconciled.

### Agonism, Institutional Imagination, and a Counter-Neoliberal Vision

In beginning to outline a politico-legal project in response to neoliberalism, primary concern must be given to the expansion of democratic possibility and our conception of the variety of matters with which democratic politics may engage and, resultantly, the variety of ways in which our institutions may be organised. This involves the expansion of democracy into areas of life from which it is excluded under neoliberalism—the workplace and broader economy, the provision and distribution of natural and vital resources, our educational institutions, and so on. This expansion challenges the neoliberal notion of the subject, *homo oeconomicus*, and aims to enliven varied methods of democratic engagement beyond the technocratic and individualised scope of neoliberalism. Crucially, these methods of democratic engagement must be *participatory*, in that they involve the active commitment of ordinary people, and *collective*, involving the organisation of citizens around common grievances. In understanding the importance of foregrounding the nature of democratic participation in a counter-neoliberal project, as opposed to advancing a strict or concrete view of institutional or policy changes, the work of legal theorist Roberto Mangabeira Unger is particularly instructive. Moreover, the work of radical democratic theorist Chantal Mouffe will be used to outline the necessity of an *agonistic*, struggle-driven approach to politics in opposition to neoliberalism. It is the contention of this article that the provision and expansion of organising rights are integral to advancing this counter-neoliberal project and are of particular use in the context of housing.

Chantal Mouffe’s conception of radical democracy is grounded fundamentally in a critique of both liberal and neoliberal approaches to democracy. Mouffe’s critique of these approaches echoes the description of the relationship between neoliberalism and democracy outlined in Part II. Mouffe states that the neoliberal conception of politics is ‘rationalist, universalist and individualist’, being concerned with individual freedom secured by universal, rational truth [2, 46]. This view is blind to what Mouffe describes as ‘the political’—the dimensions of conflict and antagonism that are constitutive of social life [47, p. 2]. Social life is defined by collective identity and the ‘we/them relation’ such that.

any type of we/them relation… becomes the site of a political antagonism. As a consequence, the political cannot be restricted to a certain type of institution… It must be conceived as a dimension that is inherent to every human society and that determines our very ontological condition [47, pp. 2–3].

This view of the political clearly explains the inability of neoliberal ideology and governance to properly comprehend or address collective tension, grievance, or prosperity. The very existence of such collective experience vitiates, to a significant degree, the neoliberal conception of humanity.

The key insight in Mouffe’s enunciation of radical, agonistic democracy is that the provision of a proper challenge to neoliberal hegemony and the pursuit of a fulsome democracy requires ‘a vibrant clash of political positions and an open conflict of interests.’ [47, p. 6] This democratic society is to be brought about through a redefinition of the political left in a way that rejects the singular approach to rationalism, individualism, and universalism advanced by neoliberalism, and radically embraces pluralism. Mouffe writes that this is a necessary precondition to ‘apprehend the multiplicity of forms of subordination that exist in social relations and to provide a framework for the articulation of different democratic struggles—around gender, race, class, sexuality, environment and others.’ [47, pp. 7–8] Political conflict, therefore, should not to be avoided in place of a liberal search for rational unity, but embraced, not for the acceleration of a predetermined set of historical developments, but embraced in and of itself.

Embracing, although not explicitly, Mouffe’s dedication to a radically democratic and experimental politics of struggle, Roberto Unger’s prescription for the problems created and intensified by neoliberalism rests upon an understanding that our society is determined neither by the ‘all-pervading underlying natural order’ of neoliberalism nor the ‘irresistible material forces’ described by Marx, but instead ‘society “is made and imagined… a human artifact”’ [62, p. 19 quoting 67, p. 1]. While this, to a degree, recalls the idealist conception of history rebuffed by Marx in OJQ, Unger’s observations usefully historicise our institutions in a way that is highly compatible with Marx and is useful in establishing the need for a revivified sense of ‘institutional experimentation’ in combatting the totalising nature of neoliberal subjectivity [69, pp. 1–4]. Neoliberalism’s limitation of democratic possibility in Unger’s work is described primarily as a result of the ‘illusion of false necessity’, that is the belief that ‘abstract institutional conceptions, like political democracy, the market economy, and a free civil society, have a single natural and necessary institutional expression.’ [69, p. 7] This illusion is based upon the ‘convergence thesis’, that the global neoliberal order is the result of ‘a convergence toward a single set of best available practices throughout the world’ and acts to reinforce the legitimacy of the political project of neoliberalism [69, p. 6]. The false logic of this thesis is present in the endism of both the neoliberal declaration of the ‘end of history’ and traditional Marxist interpretations of history.^20^

To combat the false necessity created by neoliberalism and foster radical democracy, the contrary project must be concerned primarily with how citizens engage in politics and with reinvigorating radicalism in approaches to our institutions, as opposed to advancing a particular policy set or strict political or economic agenda. In the specific context of concern in this article, greater interest should be paid to the means by which tenants can become organised than to the specific goals such tenant organisations take up.

For Unger, the ‘mobilisation’ for which this article argues can take place in the reconstruction of ‘the institutional forms of the state and of party politics, of the economy and the firm, and of civil society and its organi[s]ations.’[69, p. 164] This reconstruction contrasts neoliberalism ‘hostil[ity] to the political mobilization of the citizenry… [and] favours a persistent heightening of the level of political mobilization in society.’ [69, p. 165] In this way, a project appropriate to counter the limitation of democratic possibility under neoliberalism requires *participatory*, *collective* organising at all levels of society, across a variety of lines of conflict. Unger views it as the role of legal analysis as an ‘emancipatory social science’ to advance this process. Unger acknowledges the apparent incompatibility of rights and deepened democracy, with rights appearing as unchanging or anti-democratic phenomena as discussed in Part II. However, as will be further discussed in the following portion, Unger views certain articulations of rights, especially those in aid of radical democratic experimentation, as both compatible with and necessary for the expansion of democracy.

### The Commendation of (Organising) Rights

#### Rights and Radicalism

Unger outlines the tension between rights and democracy, specifically what both critics and defenders term ‘majoritarian democracy’.^21^ This tension views the preservation of specified rights—most traditionally through constitutional entrenchment—as withdrawing matters with respect to these rights ‘from the agenda of short-term politics’ [69, pp. 166–7]. Unger notes that constitutional entrenchment is not the only means by which this removal is achieved. ‘The cult of the constitution’ and popular conceptions of ‘natural rights’, all of which are the function of hegemony, have a similar effect of withdrawing certain matters from political contest [69, p. 167]. Unger outlines two discrete requirements for the valid removal of matters from the realm of politics through their entanglement with rights. Such matters must either concern the ‘protect[ion of] people against radical insecurities, including the risks of public and private oppression’, or such matters must ‘supply people with the economic and cultural equipment they need to define and execute their life projects’ [69, p. 167]. It is the second requirement to which the securing of organising rights most directly relates, that is that matters may be validly removed from democratic contest through the provision of rights if they provide the means by which radical democratic experimentation and the execution of a ‘life project’ may be pursued. However, it should be noted that even this is in itself an ideological, and therefore political, determination and, as Unger describes, the provision of rights grants only a ‘relative’ immunity against political change, as ‘in the end, nothing can prevent the ideas and arrangements establishing rights from remaining hostage to the practical and ideological conflicts of politics.’ [69, p. 167] Rights to collectively organise and agitate within civil society, such as those afforded most explicitly to workers through the formation of trade unions, clearly fulfil Unger’s second requirement.

Collective organisation—particularly of the oppressed, marginalised, and disempowered—is the primary means by which the radical and experimental democratic counter project previously discussed may be achieved. Such organisation, as previously stated, is *participatory* and *collective*, involving the organisation of factions of the citizenry around sites of common pain, oppression, disadvantage, and grievance. In this way, community organisations, although obviously developing their own interpretations and policy preferences along the way, do not come into existence—at least in theory—with a predetermined set of ideological commitments. The articulation of community organising espoused by Saul Alinsky, an early pioneer of community organising methodology in the United States, mirrors the vision of institutional imagination advanced by Unger. Alinsky describes community organisations as ‘politically relativistic’ in that they do not come about for the furtherance of a particular political dogma, but are founded solely on the conviction that ‘if people have the power to act, in the long run they will, most of the time, reach the right decisions.’ [3, p. 11] These organisations are, therefore, more capable of adapting to shifting circumstances and demands, and are thereby instrumental in advancing the kind of institutional imagination required in an opposition to neoliberalism.

Moreover, collective organisation of this kind is integrally agonistic and advances the kind of conflict-based politics Mouffe outlines as necessary to the counter-neoliberal project. Such organisation around sites of common grievance requires the identification of issues for which the achievement of collective power through organisation is the requisite remedy, and engagement in political struggle to win material justice. As Alinsky’s seminal expression of community organising describes, conflict (or *agonism*) and the resultant struggle for power is an integral part of organising. Alinsky writes that ‘[c]onflict is the essential core of a free and open society. If one were to project the democratic way of life in the form of a musical score, its major theme would be the harmony of dissonance.’ [3, p. 62] In this way, the promotion of organising along new and varied schisms and inequalities within society promotes a radically democratic, experimental, and agonistic politics that effectively counters the individualising and anti-democratic hegemony of neoliberalism.

#### Rights and Relationships

The role of rights discourse in the furtherance of *collective* organising also presents significant opportunities for a counter-neoliberal legal project. David Harvey’s enunciation of a collective ‘right to the city’ offers a key example of the way in which a collective right, or a set of collective rights, may act as a counter-hegemonic discursive tool in opposing the individualising rhetoric of neoliberalism [27, p. 4]. As will be further discussed, Harvey readily acknowledges that the definition and application of such a collective right is ‘itself an object of struggle, and that struggle has to proceed concomitantly with the struggle to materialize it’, mirroring the CLS critique that rights are not determinative or uncontested concepts [27, p. xv]. However, the contest inherent in the assertion of such rights is presented not as a detriment, but a benefit of such discourse. To Harvey, such a bundle of collective rights to counter neoliberalism may include any number of demands beyond traditional liberal rights [26, p. 204]. In this bundle, it is Harvey’s radicalised right to ‘political association’, infused with the primacy of class struggle in his analysis, that is of most significant concern for the purposes of this article. Moreover, the prefigurative nature of *participatory* and *collective* organising, as will be further discussed in the forthcoming section, bears what Harvey describes as the burden upon such counter-hegemonic rights discourse, by which the proposal of ‘different right to those held sacrosanct by neoliberalism carries with it… the obligation to specify alternative social process[es] within which such alternative rights can inhere.’ [26, 204] In this way, the promise of collective organising and, by extension, rights to promote organising, lies in their ability to further counter-hegemonic discourses and expand political imagination and, in so doing, the lines of political and social struggle.

Harvey describes the heavily intersectional nature of class oppression as an intensified phenomenon under neoliberalism, noting the racialised nature of lower classes throughout the Global North and the ‘increasing femini[s]ation of poverty’ [26, p. 202]. A unified language of politics and struggle is therefore required to draw together the various—and at times disparate—movements that have emerged in response to neoliberalism, from indigenous resistance to land dispossession^22^ and climate activists,^23^ to workers^24^ and LGTBQ + activists.^25^ The nature of class constitution, to Harvey, is not fixed, however, the nature of class regarding political and economic conflict, and therefore the inevitability of class struggle, is determined by the prevailing mode of production [14, pp. 279–304].

Although Harvey’s analysis seeks to a significant degree to unite these multifaceted movements behind a Marxist class politics, the substance of such movements is of lesser concern to this project than their form as organisations based around an agonistic politics of struggle. To this extent, despite its focus on class oppression, Harvey’s approach mirrors the radical democratic vision previously outlined. Moreover, Harvey’s description of the organic emergence of social and intellectual movements from determined class positions recalls the Gramscian notion of organic intellectualism and hegemony/counter-hegemony and, thereby, the counter-hegemonic role that a discourse of rights and collective organising can play.^26^

Additionally, the legal reality and discursive advancement of collective organising rights themselves provide a means of drawing together these movements and coalitions. Organising rights provide ways of validating and affirming engagement with organisations—often in the face of opposing pressure from more structurally powerful agents such as employers, landlords, or government agencies—and provide a means by which individuals may join together and, in so doing, develop collective identity. In their discussion of labour law in the service of ‘solidarity unionism’—a concept much alike the *collective*, *participatory* organising championed in this article—Staughton Lynd and Daniel Gross usefully describe these benefits as present in current protections provided in labour law. Although Lynd and Gross take a more pessimistic view of the efficacy of the law in advancing positive social change, they usefully describe that ‘[t]he best way to think of the law is as a shield, not a sword. The law is not an especially good way to change things. But it can give you some real protection as you try to change things in other ways.’ [36, p. 15].

Lynd and Gross’ emphasis on the utility of organising rights as a ‘shield’ supports the interpretation of the complication of the traditional CLS critique of right put forward by Patricia J Williams discussed in Part III. Williams’ contribution emphasises the way in which rights, although alienating to a degree, provide a formalisation and validation of identity and relationship within which vulnerable parties may engage—and possibly contest—with more structurally powerful parties. Organising rights can be seen to play this validating role by giving legal form to the engagement between, for example, individual workers or groups of workers when engaging with their employers. Where a worker may otherwise feel vulnerable to take action or raise workplace issues with their employer, the provision of organising rights to this worker allows them not to engage as an individual employee, but as a member of a designated class—‘worker’—and enjoy the protections and benefits this entails. This can allow workers to more freely engage in action and develop identity as a class, with organising rights giving validity to this way of engagement. The way in which this validating and identity-forming effect may be felt in other contexts, namely the relationship between landlords and tenants, is evident.

The view of neoliberalism in Gramscian terms—as a *hegemonic* force (per Brown) for *class power* (per Harvey)—is especially useful in understanding and expanding Honig’s conception of collective citizenship as well as the kind of class politics and social movements Harvey describes. Brown’s analysis of neoliberalism accurately depicts the ways in which neoliberalism acts to reshape subjectivity and disseminate a naturalisation and rationalisation of societal structures, however, further detailing of Gramsci’s conception of hegemony is required. Gramsci writes that these kinds of hegemonic intellectual and cultural movements emerge from class dynamics, through the creation of so-called ‘organic intellectualism’ by which social classes ‘creates together with [themselves], organically, one or more strata of intellectuals which give [them] homogeneity and an awareness of [their] own function not only in the economic but also in the social and political fields.’ [24, p. 135] In this sense, the neoliberal project of economisation is an extension of hegemonic power into a variety of previously untouched spheres of social life. Organic intellectualism, in the Gramscian sense, may arise also from the social classes that neoliberal society creates. As Harvey rightly demonstrates, neoliberal society creates and intensifies a variety of inequalities and class distinction. This intensification of class and class conflict allows for the development of organic intellectualism and a counter-hegemonic project to oppose the rationality of neoliberalism. In this way, public things may be thought of not only as forming the foundational social contract on which our society is created, but also as allowing the means by which certain classes of citizens may come to understand the world and engage with it. This further emphasises the role of public things in the development of counter-hegemonic discourse under neoliberalism, with the emergence of new forms of discourse and democratic possibility emerging from, and intensified by, inequalities created by the material and ideological conditions imposed by neoliberalism. Further, the importance of legal discourse in these analyses is of particular note, in that the law operates both as a practical mechanism for adjudicating disputes and exercising political will, and as a discourse in which citizens engage—much like the way in which neoliberalism exists as both a series of positive political and institutional prescriptions, and a mode of social, political, and economic discourse. As Brown shows, the law exists to practically affect neoliberal change, as well as define socio-political discourse within neoliberal terms. This echoes Foucault’s description that ‘[t]he economy does not purely and simply determine a juridical order… The juridical gives form to the economic, and the economic would not be what it is without the juridical.’ [19, pp. 162–3].

This analysis of neoliberalism, in combination with the views of rights discourse and organising rights as promoting organising whilst acting as a means to validate organising and connect disempowered citizens illustrates the ways in which, just as ‘public things’ in Honig’s analysis mediates our engagement with democratic life and allows for the creation of conceptions of citizenship, rights to organise along varied and intersection lines of social and political tension can mediate, shape, and accelerate our development of collective identity and our ability to engage in struggle.

#### Rights and Resistance

In further assessing the utility of organising rights, the relationship between such rights and the materialist and CLS critiques outlined in Part III will now be considered. The necessity of legal discourse in both affirming and transcending liberalism is usefully restated by CLS and labour law scholar Karl E Klare in discussing the role of law in relation to labour organising as follows:

Legal discourse shapes out beliefs about the experiences and capacities of the human species, our conceptions of justice, freedom and fulfilment, and our visions for the future. It informs our beliefs about… how we might construct the institutions through which we govern ourselves…[34, p. 1358]

The relationship between collective organising, law, and social struggle was famously explicated by Marx in his description of the evolution of laws restricting the length of the working day. For Marx, there is no material force that places a necessary limitation upon the length of a working day, but instead between the wishes of labour and capital there exists ‘an antimony, of right against right, both equally bearing the seal of the law of exchange.’ [38, p. 344] The fundamental *indeterminacy* of the work day—that is, its failure to be set by a pre-existing productive force—means that the parameters of the work day are open to contest, with Marx writing that.

[b]etween equal rights, force decides. Hence, in the history of capitalist production the establishment of a norm for the working day presents itself as a struggle over the limits of that day, a struggle between collective capital… and collective labour… [38, p. 344]

This ‘force’ is determined through collective organisation, the process by which ‘workers have to put their heads together and, as a class, compel the passing of a law, an all-powerful social barrier by which they can be [“protected”]…’ [38, p. 416] Although Marx viewed productive relationships as creating a variety of determinative processes, taken with the CLS position regarding the fundamental *indeterminacy* of the law outlined in Part III, these observations cement the integral nature of collective organising to compel the development of the law and society towards social justice.

In this way, organising rights create possibilities beyond simply the affirmation of liberal freedoms of political association. The engagement of a left-legal project should be concerned, as Williams describes, ‘not [with] the abandonment of rights language for all purposes, but [with]… becom[ing] multilingual in the semantics of evaluating rights’ [72, p. 149]. That is to say that the left-legal project should engage in what Kennedy describes as the ‘master[y] of ambivalence’—engagement with the law in spite of its fundamental indeterminacy for activist purposes [33, p. 11]. However, a project seeking to counter neoliberal hegemony should extend this engagement with the law to the active pursuit of legal protection for organising rights across new and varied social lines. Not only does the process of procuring such rights in itself open up vistas for political struggles as illustrated by CLS scholars, but these rights themselves deepen democratic engagement and function to foster collective power that then impacts and shapes the law and, most importantly, the material and social relationships that make up our society. The nature of collective or community organisations is such that they in themselves allow for the development of collective power and, in their structure, methods, and practices, are able to *prefigure* the kind of institutions their members wish to see established. This analysis of the revolutionary potential of organisations is at variance with traditional Marxist interpretations of the role of organisations, in that organisations require the existence of social and political tension in order to validate themselves and therefore are incapable of fully overcoming these tensions. Applying this criticism to the trade unions of his time, Marx remarked that trade unions ‘[limit] themselves to a guerrilla war against the effects of the existing system, instead of simultaneously trying to change it’ [41, 122]. Mouffe’s insight regarding the necessity of agonism to democratic life and Unger’s anti-necessitarian vision, however, can be seen to address Marx’s critique of organising in that organising is an end to be pursued in and of itself, not purely as an instrument for the achievement of a particular political objective.

Moreover, the internal structure of organisations are able to adapt to the changing circumstances of the sites of common grievance around which they are formed, and present an alternative vision and structure for social institutions. For example, historical instances of tenant organising have served a dual role in providing immediate means for negotiation and bargaining with landlords, and have also been seen—through the provision of community employment and food services, community protection programs, and other activism not directly related to their status as tenants—to ‘articulate… a “right to the city”… that was richer and more communal than the narrow self-interest expected of them by market-based urban governance’ in the face of crises in their communities [30, pp. 400–3]. The existence of such democratic organisation allows for both the exemplification of new forms of social arrangement and the creation of the practical means by which existing arrangements may be supplanted through the development of collective power. In its most evident form, this can be seen in community organisations, in the face of crises, taking initiative to purchase the very factories or houses with whose previous owners they were bargaining, and instituting structures of collective ownership. This process is noted by Lynd and Gross in their conception of ‘solidarity unionism’ that seeks to not only protect workers in their current relations with employers, but take such positive steps to advance alternative structures of firm ownership and workplace relations that are more egalitarian and democratic [36, pp. 73, 95]. The guarantee of a ‘right of first refusal’ for workers to buy factories that seek to ‘off-shore’ their workforce is gaining political support in many deindustrialising nations and can be seen as a counter to neoliberal globalisation that repurposes an extant concept in corporate and commercial law for activist purposes [36, p. 95. See 82, 89]. The extension of such reform to tenant engagement with corporate landlords, for example, presents further opportunity. Again, the existing structure of labour and industrial relations law provides a forceful illustration of the ways in which such rights frameworks already exist, although imperfectly.

## The Possibility of the Commons: Tenants’ Rights to Organise

The foregoing discussion requires now the consideration of what practical legal reforms should be championed in the service of such organising. In this regard, the emergent scholarship in the area of ‘movement law’ is of particular use. Movement law scholarship seeks to expand upon and radicalise traditional scholarship regarding law and social movements by developing an approach that is ‘grounded in solidarity, accountability, and engagement with grassroots organi[s]ing and left social movements’ [2, p. 821]. Movement law seeks to address both the discursive and instrumental aspects of the law as it both hinders and advances organising. The duality of this approach will be considered throughout the following concluding reform considerations. Moreover, the movement law approach can be seen to synthesise the views on the relationship between law and political struggle previously outlined by utilising the ability of legal discourse, properly deployed, to counter the hegemonic power of neoliberalism.

First, as discussed in Part III, the role of the law in providing a suitable ‘battleground’ upon which organisations may engage is integral. This process is described by Benjamin I Sachs and Kate Andreas as ‘framing’, by which the legal presentation of legal rights to organise creates—both discursively and materially—the space for such organisations to challenge and expand politico-legal discourse and fight for the preservation and expansion of other rights beyond the limits of neoliberalism. In the specific context under consideration—the collective organisation of tenants—Sachs and Andreas argue that statutory rights and standards pertaining to housing, such as those that already exist under residential tenancies legislation in various Australian jurisdictions,^27^ should be presented in legislation alongside.

the right to organi[s]e collectively to enforce those standards and to achieve greater substantive protections in the future… affirm[ing] a right to adequate and sustainable housing, grant[ing] tenants the right to just-cause eviction, and also grant[ing] tenants a right to organi[s]e unions [4, p. 592].

Such presentation of organising rights clearly presents collective organisation as the means to the full achievement and enjoyment of traditional rights in the housing sphere, whilst providing the potential for the furtherance of what Akbar describes as ‘radical imagination’, that is the ability of collective organisations and social movements to further in lawmaking and legal analysis conceptions of our society’s institutions in which ‘the scale of deep critique is matched with a scale of grand vision’ for radical reform [1, p. 412]. This radical imagination clearly illustrates the kind of radical democratic vision required to step outside the hegemony of neoliberalism.

The utility of framing is exemplified in the interaction between tenants’ rights and the *Victorian Charter of Human Rights and Responsibilities Act 2006* (Vic) (‘*Charter*’). Although the *Charter* does not provide any substantive guarantee of housing, it has been used by the Victorian Civil and Administrative Tribunal (‘Tribunal’) to limit ‘no cause’ evictions.^28^ Such utilisation of the *Charter* allows for intervention by the Tribunal to prevent significant injustice befalling tenants in a way that generally accords with liberal jurisprudence and acknowledges ‘the extreme consequences of an eviction [with respect to human rights], particularly when this results in a person being made homeless… and highlight[ing] the lack of procedural protections for a tenant when eviction is based on a no-reason notice to vacate.’ [60, p. 83] However, this approach does not in itself remedy the fundamental differential of power between landlords and tenants, and does not provide any specific guarantee of housing [91, p. 3]. In this way the Tribunal’s reasoning can be seen as the kind of liberal political victory of which the CLS tradition warns we should be appreciative, whilst readily acknowledging such reasoning’s insufficiency and offering radical critique. The rights protections the *Charter* affords could, therefore, be extended and enhanced—or, in other words, these rights could be *radicalised*—through the kind of legislative framing advanced by Sachs and Andreas, especially given the prominence of tenant unionism in Victoria [92, p. 2]. This framing—creating space for radical contest regarding the terms of political, economic, and social life by presenting the aforementioned liberal rights alongside rights to protect tenant organising—can go some way towards metamorphosing such rights from their present, liberal form to something far more radical.

Second, for the purposes of this analysis, Sachs and Andreas’ acknowledgment of the law’s role in creating and redistributing resources, and its application to organising, is of particular importance. Early literature regarding tenant unionism notes the significant hurdles faced in the establishment and sustainment of tenants’ unions due to the significant effort and financial outlay required, as well as issues pertaining to funding to maintain and expand the work of such organisations [61, p. 1369]. Sachs and Andreas draw upon a significant amount of organising and social movement literature that indicates the unique precarity of grassroots organising amongst low-income groups in this regard [4, pp. 595–8]. This accords with the author’s own experience in tenant and low-income organising, in which tenants and low-income groups experience a unique array of social, financial, and emotional strains that limit engagement with organisations that are integrally linked with, and resultant from, the very conditions sought to be remedied by organising. Sachs and Andreas position legal mechanisms—such as those of taxation and contract law—as uniquely able to promote the funding of tenant organisations. This occurs, first, through the provision of tax-deductable funding options as well as the provision of state subsidies. Further, the law can also provide for a variety of self-funding mechanisms, namely the facilitation of the payment of ‘dues’ akin to those utilised by trade unions, through payment arrangements by which wages or rent payments are garnished to finance union membership [4, pp. 595–8]. Sachs and Andreas also outline ‘cost-shifting’ mechanisms which seek to use the law to ‘shift costs to the entity around which the social-movement organi[s]ation is organi[s]ing… [by] require[ing] those entities to pay for a portion of the organi[s]ing activity.’ [4, p. 605] Cost-shifting and state funding mechanisms, despite their use in establishing organisations, may in practice somewhat undermine the independence of such organisations, especially once the initial cost of establishing organisations is dealt with, making organisation financially dependent upon those against whom they are organising and, to a certain degree, fall victim to Marx’s critique of trade unions previously discussed and dampen the potential of these organisations to agitate for more radical change.

Further, legal mechanisms can be utilised to create ‘free spaces’ within which tenants’ unions can organise [4, p. 613]. The utility of such protections is made clear through analogy to existing ‘right of entry’ protections for trade unions.^29^ This can involve union officials inspecting the condition of a property as a means to protect the rights of their tenants, however it can also extend to a union’s ability to organise within shared spaces, for example a walkway or communal area in an apartment building. This creates physical space within which organising is legally protected to occur. Utilising Honig’s analysis of ‘public things’ discussed in Part II and IV, this free access of organisations to communal spaces allows for physical geographies and objects common to tenants to be transformed into sites of organisation and objects of mediation through which collective, democratic identity can take shape.

Third, echoing this use of free spaces in the pursuit of organisation and the creation of collective identity, Sachs and Andreas note the significant ways in which legal mechanisms can act to remove ‘barriers to participation’ in tenant organisations [4, p. 620]. This can be seen in the way in which, as discussed in Part IV drawing upon Williams’ articulation of rights as both a ‘shield’ and a ‘sword’, organising rights can protect tenants from retributive action by landlords in their attempts to unionise, and can provide legal legitimacy to tenants’ organisations and thereby, through the distance and protection afforded by legal discourse, empower tenants to take collective action. In this way, the law can play a constitutive role in the formation of tenants’ unions, providing legitimacy to such organisations and a framework upon which they may be built. This view of law’s utility acknowledges its role in validating neoliberal and counter-neoliberal power structures, as well as the political utility of the legal contest found in attempting to transition between such structures.

Finally, Sachs and Andreas further support the overall contention of this article that organising rights can serve the furtherance of ‘contestation and disruption’ in the protection of collective action such as protest and rent strikes [4, p. 627]. The legal protection of such collective action, although itself not fundamentally transgressing the landlord-tenant relationship—just as industrial action protections do not *fundamentally* transgress the worker-owner relationship—does act to revivify the landlord-tenant relationship as a site of *agonism* in our politics and economy. This epitomises the thrust of CLS legal activism and provides the means by which conflict can be spurred that may expand and transcend the limits of neoliberalism, not only through the expansion of democratic possibility and opposition to neoliberal doctrine, but through the reinforcement of such discursive victories with material advancements in favour of tenants and, more broadly, those disenfranchised and disempowered by the march of neoliberalism.

## Conclusion

The characterisation of the constitutive role of law and legal discourse in the neoliberal project and its remaking of democratic subjectivity outlined herein makes clear, with reference to the work of Unger and Mouffe, the necessity of a radical and democratic counter project that promotes *participatory*, *collective* organisation to open new veins of agonism and democratic contest throughout society. In particular, Honig’s conception of ‘public things’ as ways of mediating civic experience and creating civic and democratic identity is integral in establishing the foundations of such a counter project. As put forward in the analysis of law and social change and, in particular, the left-legal critique of rights, explored in Parts III and IV, rights, both discursively and instrumentally, are integral to this project.

As outlined in Part IV.II.I, organising rights are indispensable to a *collective*, *participatory* radical democratic political and legal project to counter neoliberalism. Such organisations are inherently agonistic and aim to ‘radicalise’ areas of life and society that have been depoliticised through the neoliberal project. Moreover, drawing upon both Unger and Alinsky, the role of such organisations in advancing the kind of radical imagination required in this new politics is clear. This political role of *participatory*, *collective* organising was returned to in Part IV.II.III as a synthesis of the materialist critique of law and, particularly, of rights under liberalism, and the CLS approach to the instrumentality and imperfection of law as a tool for activism. Part IV.II.II addressed rights discourse as a counter-hegemonic tool and, again drawing upon the perspective of scholars in the CLS tradition, discussed the role of rights discourse as both a sword and shield for providing ways, rhetorically and materially, in which people may draw together and organise, and way in which organisations of oppressed and marginalised people may relate to the groups, institutions, and structures they wish to challenge. Further, the analysis of neoliberalism put forward in Part II emphasises the ways in which, just as ‘public things’ in Honig’s analysis mediate our engagement with democratic life and allow for the creation of conceptions of citizenship, rights to organise along varied and intersecting lines of social and political tension can mediate, shape, and accelerate our development of collective identity and our ability to engage in struggle. Not only are organising rights, properly deployed, instrumental legal tools for promoting the kinds of *collective*, *participatory* organising necessary to form a counter-neoliberal project and revivify democratic life—as explored previously in Part V—but such rights also serve an integral discursive function. The role of legal discourse as a constitutive part of social life is aptly utilised through the discursive qualities of rights discourse, properly charactered in toto as the ‘semiotic indeterminacy’ of rights, in the building of a counter-neoliberal political and legal project. In her analysis of the rhetoric utilised by the Serbian *Otpor* movement, Jessica Greenberg notes that such movements ‘rel[y] of semiotically flexible repertoires that [can] both unify the movement as a whole and be taken up and adapted to local conditions.’ [25, p. 379] As the previous analysis makes clear, Greenberg’s enunciation of semiotic flexibility in the *Optor* movement is generally applicable to the rhetorical utility of rights discourse heretofore advocated. Organising rights present an opportunity for the creation, both rhetorically and legally, of radically democratic organisations that may not only rise to address the crises of neoliberalism, but prefigure and begin to establish a world beyond its bounds.

## Footnotes

1 David Graeber, ‘After the Pandemic, We Can’t Go Back to Sleep’, *Jacobin *(online), 4 March 2021 <https://jacobinmag.com/2021/03/david-graeber-posthumous-essay-pandemic>.

2 See, eg, [75, s 8(1)(b)].

3 See, eg, [75, s 8(1)(d); 76, s 24(2)(a)].

4 See, eg, [53, p. 100; 64, pp. 448–57].

5 See [54].

6 See the work of Wendy Brown discussed in Part II.

7 See, eg, [8, 32, 65].

8 See, eg, [57, p. 7].

9 See, eg, [4, p. 586]. For an Australian articulation of ‘movement law’ scholarship, see [88].

10 Financialization refers to ‘[t]he process whereby finance capital and financial institutions account for a greater share of all economic activity’: [50]. As discussed further in Part II, Brown takes an expanded definition of financialization, considering the ways in which neoliberalism reshapes civil and political subjectivity.

11 It is noted, however, that more fulsome consideration of the policy proposals that flow from such a program is beyond the scope of the initial project this article represents.

12 See generally [10].

13 See, eg, [6, pp. 445–50; 42, pp. 141–2; 63].

14 For a further explanation of the context and contested history of the ‘New Left’ see, eg, [43, p. 1]. In 1962… a cadre of student activists began the New Left… under the somewhat impertinent notion that they might set forth “an agenda for a generation.” They were influenced by a wide array of sources, including… critical sociology… French existentialism, and theories of participatory democracy derived from the civil rights movement, as well as less obviously political sources—Mad magazine, Beat poetry, the hipster ethos of the “White Negro,” and left-wing folk music. Although they were not communist sympathizers, they refused to declare themselves anticommunist, thereby distinguishing themselves from parts of the Old Left. [Leading]… lives of “principled nonconformity,” devoted themselves to “secular ideals of social justice,” and (like all good liberal reformers) exhibited great faith in the transforming potential of marches, meetings, and mimeograph machines. [43, p. 3].

15 See [21].

16 See, eg, [48; 49].

17 See above Part III(A).

18 For discussion of a similar perspective expressed in the Australian context, see [43].

19 See, eg *Fair Work Act* (Cth) 2009s 346 (a) and (b): A person must not take adverse action against another person because the other person:

- Is or is not, or was or was not, an officer or member of an industrial association; or.
- Engages, or has at any time engaged or proposed to engage, in industrial activity within the meaning of paragraph 347(a) or (b).

*Fair Work Act* (Cth) 2009s 772(1)(b), (d) and (e):

1. An employer must not terminate an employee’s employment for one or more of the following reasons, or for reasons including one or more of the following reasons:

(b) trade union membership or participation in trade union activities outside working hours or, with the employer’s consent, during working hours; … (d) seeking office as, or acting or having acted in the capacity of, a representative of employees; (e) the filing of a complaint, or the participation in proceedings, against an employer involving alleged violation of laws or regulations or recourse to competent administrative authorities; …

20 See, eg, [5; 20].

21 See generally [70].

22 See, eg, [24, pp. 849–52].

23 See, eg, [84].

24 See, eg, [16, pp. 17–8].

25 See, eg, [14, pp. 279–304].

26 It should be noted that descriptions of neoliberal hegemony are not normative in the sense that hegemony itself, and the role of law in its maintenance and furtherance, are the issue to be combatted by any left-legal project. The understanding of the hegemonic power of law to be advanced is descriptive. The law maintains and furthers prevailing social powers, whilst also creating space of contest and radicalism—as per the CLS critique. Hegemony is not necessarily the political ill to be remedies, but the quality of hegemony under the prevailing social structure.

27 See, eg, [78; 79; 80].

28 See, eg, [74].

29 See [77, ss 481–521].
